# Delivery of nutritional management services to people with amyotrophic lateral sclerosis (ALS)

**DOI:** 10.1080/21678421.2021.1874991

**Published:** 2021-01-28

**Authors:** Vanessa Halliday, Nicolò Zarotti, Elizabeth Coates, Alexander McGeachan, Isobel Williams, Sean White, Daniel Beever, Paul Norman, Sarah Gonzalez, Gemma Hackney, Naseeb Ezaydi, Theocharis Stavroulakis, Mike Bradburn, Christopher McDermott

**Affiliations:** 1 School of Health and Related Research (ScHARR), The University of Sheffield, Sheffield, UK; 2 Sheffield Institute for Translational Neuroscience (SITraN), The University of Sheffield, Sheffield, UK; 3 Department of Psychology, The University of Sheffield, Sheffield, UK; 4 Sheffield Teaching Hospitals NHS Foundation Trust, Sheffield, UK

**Keywords:** Nutrition management, health services, mixed methods, amyotrophic lateral sclerosis, motor neuron disease

## Abstract

Abstract

Objectives

The aim of this study was to investigate how nutritional management services for people with Amyotrophic Lateral Sclerosis (pwALS) are structured in the UK, in order to gain insight into current practice and identify key barriers and enablers to delivering and providing services. *Methods*: A three-part, sequential mixed-methods study was conducted that comprised (i) a thematic analysis of data from five focus groups (with 47 ALS health professionals from 41 UK organizations and four service user representatives), (ii) a nationwide cross-sectional survey (281 ALS healthcare professionals) and (iii) a freedom of information request (251 organizations). *Results*: UK nutritional management services for pwALS are coordinated from specialist (*n* = 22) and non-specialist care centers (*n* = 89), with national variability in the organization and delivery of services. Multidisciplinary working was highlighted to facilitate the coordination of nutritional care. However, the need to provide evidence-based continuing education for HCPs was evident. Overall, the lack of clear guidelines on the nutritional management of people with ALS was identified as a key barrier to the delivery of effective nutritional care, as was the lack of transparency and consistency in the commissioning of nutritional services. Further concerns over the timeliness of the dietetic intervention and equity of access and provision were raised. *Conclusions*: Our findings suggest that development of guidelines for nutritional management, particularly at diagnosis and pre-gastrostomy, could drive standardization of high quality nutritional care for pwALS. Such guidance has the potential to reduce inequalities in geographical provision by providing clarity for those commissioning specialist nutrition services.

## Introduction

Weight loss is commonly reported in people with amyotrophic lateral sclerosis (pwALS) with a higher body mass index (BMI) and obesity believed to have beneficial prognostic effects (1–3). Furthermore, prevention of weight loss can positively impact quality of life, particularly with regards to reducing levels of fatigue (4).

Despite the complex etiology of malnutrition, there is evidence that provision of nutritional support is of benefit (4–6). Although international ALS nutritional guidelines identify the importance of addressing dysphagia and the timing and method of gastrostomy insertion (7–10), guidance on nutritional management with regard to assessment of nutritional status, appropriate dietary intake or oral nutritional supplementation is lacking. There is a dearth of evidence-based guidance specific to the structure and delivery of nutritional management services for newly diagnosed pwALS. Moreover, surveys of healthcare professionals (HCPs) have highlighted the lack of evidence and knowledge regarding nutritional management of ALS as a concern (11–13).

In the UK, nutritional management is a research priority for pwALS (14). An improved understanding of the organization of nutritional management services, from diagnosis and throughout the disease course, could guide interventions, thus preventing the negative consequences of weight loss and subsequent malnutrition. The aim of this study was to investigate how nutritional management services for pwALS are structured in the UK, to explore current practice and identify barriers and enablers to provision.

## Methods

### Study design

A mixed-methods approach was chosen due to the complexity of the issue under investigation, involving various national stakeholders. Qualitative data was used to provide contextual understanding to quantitative findings and to facilitate investigation of different aspects of nutritional management services. The mixed-methods approach has been found to be powerful in health services research (15,16).

This was a three-part exploratory sequential study comprising: (i) focus groups (FGs) with ALS HCPs in different locations across the UK, (ii) a nationwide cross sectional survey of ALS HCPs and (iii) freedom of information requests sent to UK healthcare organizations.

### Part 1 qualitative focus groups

#### Participants

UK healthcare professionals involved in ALS care and service user representatives were invited to participate in focus groups. A range of organizations were contacted including the Motor Neurone Disease Association (MNDA), MND Scotland, British Dietetic Association (BDA), UK Motor Neurone Disease Clinical Studies Group, and Sheffield Motor Neurone Disorders Research Advisory Group. In addition, MND Care Centers, Clinical Commissioning Groups (CCGs), and MND Regional Care Development Advisers were approached. Invitation emails were distributed to contacts, and study information was advertised on Twitter. Convenience sampling methods were used, whereby eligible and potentially interested participants were asked to opt-in based on their availability to attend one of the focus groups, and to maximize sample variation in terms of clinical specialties, job roles and geographical locations.

#### Data collection

Focus groups were conducted in June 2018 and held at easily accessible non-NHS sites throughout the UK. Each focus group discussion was divided into three sections. In section one, the groups were asked “How important is it to provide nutritional advice and support to pwALS?” to encourage open discussion. Section two involved a small-group elicitation exercise whereby participants were asked to record summary information to allow the study team to produce descriptive accounts of commissioning and guidance, service structure, and interventions to inform the survey design. Section three was an exploratory discussion of barriers and enablers to delivering nutritional management services. The focus group discussions from sections one and three were audio recorded. Two members of the research team (EC, AM) facilitated the FGs, with support from others (IW, DB, GH).

#### Analysis

Recordings were transcribed verbatim and analyzed thematically (17). This began with data familiarization, to develop a preliminary list of codes. Following multiple revisions by three team members (NZ, AM, LC), codes were distilled into four overarching and distinctive themes on which all the members of the team agreed, two of which are presented here (the other two themes are presented elsewhere (18)).

### Part 2 cross-sectional survey

The survey, hosted online via Qualtrics, included a set of questions structured under eight headings: (1) demographic information about participant role and care location, (2) involvement with pwALS, (3) multidisciplinary team (MDT) working, (4) nutrition knowledge and skills, (5) nutrition and dietetic services, (6) nutritional screening, (7) nutritional management and (8) commissioning and funding of ALS services. As dietetic practice was of specific interest, additional questions related to nutritional assessment and treatment of patients were asked through conditional branching (findings reported elsewhere (19)). The majority of questions quantitatively explored the knowledge and attitudes of participants. Prior to dissemination, the survey was piloted locally with nine healthcare professionals.

#### Participants

Healthcare professionals working in the UK with current or recent involvement in ALS clinical or community care were eligible to participate. The survey was distributed electronically to known contacts and via gatekeepers at UK MND Care Centers, NHS Trusts, the MNDA, and through profession specific networks (National Nurses Nutrition Group, and specialist groups of the BDA: Neurosciences Specialist Group, and Parenteral and Enteral Nutrition Group). It was also publicized via social media, websites, and newsletters. Focus group participants who consented to be contacted about future research were sent the survey directly.

A snowballing technique was used to distribute the survey to HCPs across geographical areas. To maximize the response rate, two reminders and an incentive prize draw were included. The survey was open between 19 September and 14 November 2018.

### Data analysis

Findings were analyzed in SPSS and summarized descriptively.

### Part 3 freedom of information requests

Freedom of Information (FOI) teams at UK NHS Hospital Trusts and Clinical Commissioning Groups (CCGs) were contacted via email, requesting information about services provided for pwALS. The questions addressed the size, structure, and location of the ALS service, dietetic provision, and commissioning of ALS nutrition services. Prior to dissemination, the FOI questions were piloted locally.

#### Ethics

Ethical approval for this study was granted by the Research Ethics Committee of the School of Health and Related Research at the University of Sheffield (ref: 018781), and governance approval was granted by the Health Research Authority (ref: 245296). All focus group participants provided written informed consent. The survey included a participant information section and questions documenting consent to participate.

## Results

### Sample characteristics

There were 51 participants across the five focus groups. The number of participants at each focus group varied from seven to a maximum of 13. The mean duration of the discussions was 60 minutes (range from 55 to 65 minutes).

In total, 281 participants completed the survey. Participants had been in their post for a mean of seven years (SD = 6.2), with 9.8 years mean experience (SD = 7.4) working with pwALS. The majority (67.8%) reported that patients with ALS were less than 20% of their caseload.

Of the 433 organizations contacted, 379 (87.5%) responded within the seven-week deadline. The FOI request was relevant for 251 (66.2%) of these organizations, 109 (43.4%) of which were Health Boards or NHS Trusts that provided healthcare services for pwALS. Twenty-two (8.8%) stated that they were a specialist care center.

Participant details are shown in Tables 1 and 2.

**Table 1:** Participant professions.

	Focus groups *n* (%)	Survey *n* (%)
Dietician	25 (49%)	130 (46%)
Nurse	11 (22%)	56 (20%)
Speech and language therapist	6 (12%)	35 (12%)
Service user	4 (8%)	0
MND coordinator	2 (4%)	4 (1%)
Doctor	2 (4%)	34 (12%)
Physiotherapist	1 (2%)	11 (4%)
Occupational therapist	0	10 (4%)
Psychologist	0	1 (<1%)
Total	51	281

**Table 2:** Summary of organizations responding to FOI request.

Organization	Response frequency (%) *n* = 251	Number that commission or provide healthcare services for pwALS
Clinical commissioning groups	114 (45%)	14
London	18	1
Midlands and East	43	6
North	28	3
South East	19	2
South West	6	2
Health boards	21 (8%)	20
Northern Ireland	5	5
Scotland	10	9
Wales	6	6
NHS Trusts in England	114 (45%)	89
London	18	15
Midlands and East	29	22
North	34	22
South East	17	16
South West	16	14
Non-NHS	2 (<1%)	2
London	1	1
Nationwide	1	1

### Identified themes

Two overarching and distinctive themes in relation to the organization of ALS services in the UK were identified across the three parts of the study. The first theme concerned the determinants of quality healthcare and issues related to timely and effective care. The second concerned the importance of organization and team working, and how improving communication at different organizational levels could improve the delivery of effective nutritional care. The results have been integrated into a narrative to describe each theme, drawing on the qualitative themes from the focus groups and triangulated with data from the survey and FOI requests. Table 3 shows a selection of illustrative quotes from the focus groups to support each of the sub-themes. Tables 4 and 5 show summary statistics from the survey and FOI request, respectively.

**Table 3:** Illustrative quotes from focus groups.

Theme	Illustrative quote(s)
Determinants of quality care	
MND dietetic services	“I think the earlier we can get in there and help provide reassurance and advice and guidance, then I think we can do a better job. And the sooner we get to know people and provide that advice earlier I think do better toward the end when things can get more complicated, cos you’ve got the relationship, you’ve got the knowledge of the patient, the family. The sooner you can start to develop that, the more appropriate everything you do is gonna be.” – Focus group 1 “As dieticians we end up getting involved at crisis point.” – Focus group 3
Setting of ALS care delivery	“Community service is so important because it’s no point bringing someone into an appointment, that’s not what you do at home that, you know, I want to see where you are sitting and how you’re sitting and how much your food is given and what else is going on. So you can’t really judge how someone’s physically managing.” – Focus Group 3
Nutrition knowledge and skills	“I’m not looking for anything that is prescriptive, but it would be nice to have a bit more guidance, a bit more evidence behind things.” – Focus Group 2 “Part of that [nutritional management] is developing the relationship, isn’t it? If you’ve got a relationship, trust with your patient, that really, really helps. But it can take time to kind of develop that, can’t it […] because you can’t have those difficult conversations when you’ve just met.“ – Focus Group 3 “like a sort of national tool kit that can be adopted by any trust” – Focus Group 2
Commissioning of ALS services	“Yeah, there’s a huge gap for the whole of neurology services for nutrition.” – Focus Group 1 “There is a certain amount of discretion where a manager will say ‘we’re not actually funded to do this, but I want you to go out and I want you to go and see these people’, and that is something that enables the service. In other departments they might say ‘we’re not funded for that, you’re not seeing those people’.” – Focus Group 1 “Part of the issue is that the tariff… depending on where you are, the tariff is being based at the clinic tariff, and that’s just whether you’re hospital or community for that matter, and actually it’s just illogical, given the complexity of the patients. So part of the money would come about if it was commissioned as a specialist clinic and specialist service.” – Focus Group 3
Organization and MDT working	“Having a specific MND MDT team is a huge advantage, because it’s not just the dietician that’s really key for nutritional support to management, it’s the whole of the team.” – Focus Group 2

**Table 4:** Summary of findings from survey.

	Responses	*n* (%)
Involvement with ALS services		
Do you currently provide care to people with motor neurone disease (MND)?		281
	Yes	255 (90.7)
	No, but I have in the past	23 (8.2)
	No	3 (1.1)
Approximately, what percentage of your total caseload at present are patient with MND?		255
	0–20%	173 (67.8)
	21–40%	42 (16.5)
	41–60%	6 (2.4)
	61–80%	6 (2.4)
	81–100%	28 (10.9)
ALS patients represent 20% or less of caseload based on profession		
	Dieticians (*n* = 130)	102 (78.5)
	Doctors (*n* = 34)	24 (70.6)
	Nurses (*n* = 56)	32 (57.1)
	Occupational therapists (*n* = 10)	6 (60)
	Physiotherapists (*n* = 11)	8 (72.7)
	Speech and language therapists (*n* = 35)	18 (51.4)
Where is/was that care delivered?		278
	Patient’s homes	185 (66.6)
	Palliative care centers/hospices	115 (41.3)
	Hospital inpatients services	113 (40.6)
	Hospital outpatients specialist ALS clinics	73 (26.2)
	Community GP clinics	10 (3.6)
	Hospital outpatients general clinics	45 (16.1)
	Hospital outpatients neurology clinics	17 (6.1)
MND multidisciplinary team (MDT) working		
Do you consider yourself to work as part of a MND MDT?		281
	Yes	214 (76.1)
	Unsure	10 (3.6)
	No	57 (20.3)
If no, is there a MND MDT within your organization?		57
	Yes	35 (61.4)
	No/Unsure	22 (38.6)
How do you interact with the MND MDT?		214
	Regular attendance at MDT meetings	118 (55.1)
	Regular attendance of specialist clinics	56 (26.2)
	Written/verbal communication when required	139 (64.9)
How effective is communication within MDT regarding nutritional management of MND patients?		281
	Not at all effective	4 (1.4)
	Slightly effective	24 (8.5)
	Moderately effective	104 (37)
	Very effective	116 (41.3)
	Extremely effective	33 (11.7)
How well coordinated is the approach to nutritional management within your locality?		281
	Uncoordinated	20 (7.1)
	Not very well coordinated	38 (13.5)
	Moderately well-coordinated	114 (40.6)
	Very well coordinated	99 (35.2)
	Extremely well-coordinated	10 (3.6)
Nutrition knowledge and skills		
Have you ever provided nutritional advice to MND patients or their carers?		281
	Yes	246 (87.5)
	No	35 (12.5)
If yes, do you base your nutritional advice on set guidelines or standards?		246
	Yes	143 (58.1)
	No/Unsure	103 (41.9)
Which guidelines or standards do you use?		143
	Motor Neurone Disease Association (MNDA)	118 (82.5)
	National Institute for Health and Care Excellence (NICE)	116 (81.1)
	Parenteral and Enteral Nutrition Group (PENG)	83 (58)
	British Association for Parenteral and Enteral Nutrition (BAPEN)	69 (48.3)
	Locally developed NHS Trust guidelines	63 (44.1)
	European Society for Enteral and Parenteral Nutrition (ESPEN)	25 (17.4)
How do you rate your level of knowledge of nutritional issues in MND?		281
	Very poor/Poor	10 (3.6)
	Fair/Good/Excellent	271 (96.4)
How satisfied are you with your level of knowledge of nutritional issues in MND?		281
	Not at all satisfied	7 (2.5)
	Slightly satisfied	20 (7.1)
	Moderately satisfied	123 (43.8)
	Very satisfied	111 (39.5)
	Extremely satisfied	20 (7.1)
Those who reported moderate satisfaction with level of knowledge of nutritional issues in MND, based on profession		
	Doctors (*n* = 34)	18 (52.9)
	Dieticians (*n* = 130)	47 (36.1)
	Nurses (*n = 56)*	25 (44.6)
	Occupational therapists (*n* = 10)	6 (60)
	Physiotherapists (*n* = 11)	7 (63.6)
	Speech and language therapists (*n* = 35)	20 (57.1)
Funding of ALS services		
To what extent do you agree with the following statement: “there is sufficient funding for the nutritional management of patients with MND in your locality?”		281
	Strongly agree/agree	65 (23.1)
	Strongly disagree/disagree	109 (38.8)
	Neither agree nor disagree	107 (38)
What level of priority do you believe is given to commissioning services that support the nutritional management of patients living with MND?		281
	Medium to very high priority	150 (53.4)
	Low priority	114 (40.6)
	Very low priority	17 (6)

**Table 5:** Findings from FOI request.

	Responses	*n* (%)
Of the 433 organizations contacted		379
	FOI request was relevant	251 (66.2)
	Commission or provide healthcare services for pwALS	125 (49.9)
Motor Neurone Disease (MND) Services		
Is your NHS organization a specialist care center for MND?		109
	Yes	22 (20.2)
	No	87 (79.8)
In your organization, are patients with MND seen in specialist clinics?		125
	Yes	49 (39.2)
	No	76 (60.8)
If yes, how often are specialist clinics held?		49
	Weekly	10 (20.4)
	Every 2–4 weeks	19 (38.8)
	2–4 months or less frequently	18 (36.8)
	Not known	2 (4)
If yes, how often are patients routinely reviewed in MND specialist clinics?		52
	Every 2–3 months	26 (50)
	Determined by clinical need	15 (28.8)
	No formal follow up	1 (1.9)
	Every 3–6 months	10 (19.2)
MND Healthcare Team		
Who leads the MND services in your organization?		89
	Consultant neurologist	41 (46.1)
	Neuro/rehab team	9 (10.1)
	Palliative medicine consultant	7 (7.8)
	Specialist nurse/matron	7 (7.8)
	ALS coordinator / advisor	4 (4.5)
	No specific lead	21 (23.6)
Is there a multidisciplinary team (MDT) providing care for ALS patients?		97
	Yes	71 (73.2)
	No	26 (26.8)
Location of MDT		71
	In specialist care centers	21 (29.6)
	In non-specialist care centers	50 (70.4)
If yes, which professional roles are members of the MDT**?**		71
	Occupational therapist	66 (93)
	Dietician	56 (79)
	Physiotherapist	50 (70)
	ALS/neurology specialist nurse	42 (59)
	Speech and language therapist	39 (55)
	Neurology consultant	38 (54)
	Social worker/benefits advisor	29 (41)
	Palliative medicine consultant	23 (32)
	Psychologist	16 (23)
	Respiratory consultant	15 (21)
	Hospice representation	14 (20)
	ALS coordinator/advisor	11 (16)
	Palliative medicine nurse	10 (14)
	Community nurse	9 (13)
	Gastroenterology consultant	4 (6)
	MNDA representation	4 (6)
	Respiratory physiologist/technician	4 (6)
	Rehabilitation consultant	4 (6)
	Wheelchair advisor	4 (6)
	GP	2 (3)
	Orthotist	2 (3)
	Nutrition nurse	1 (1)
	NIV practitioner	1 (1)
	Service user	1 (1)
Do you have posts specifically funded for ALS care?		111
	Yes	38 (34.2)
	No	73 (65.8)
If yes, what type of posts are these?		38
	Specialist nurse	24 (63.1)
	ALS coordinator	20 (52.7)
	Clinical lead	8 (21)
	Dietician	7 (18.4)
	Psychologist	2 (5.2)
Commissioning of MND Nutrition Services		
Where does the funding come from for MND services in your locality?		202
	Clinical commissioning groups	107 (52.9)
	Charities	8 (4)
	No specific funding	15 (7.4)
	NHS England, Health Boards, NHS Trusts and specialist commissioning	55 (27.2)
	Information not available	17 (8.4)
Where does the funding come from for MND nutritional services in your locality?		143
	Clinical commissioning groups	86 (60.1)
	NHS England	21 (14.7)
	Department of Health	1 (0.7)
	Scottish Government	3 (2.1)
	County Council	1 (0.7)
	No specific funding	31 (21.7)

### Determinants of quality healthcare

This study identified many factors that influence how healthcare services interact with ALS patients at opportune moments to impact positively upon nutritional status. The complexity of nutritional management in ALS was acknowledged by participants across focus groups.

#### Access to services

In the focus groups, it was apparent that access to many services and treatments for pwALS with nutritional issues varied between geographical areas and NHS Trusts. Participants believed that this created heterogeneity in care and barriers to delivering optimal nutritional management, to the point that access to services was described as “pot luck.”

Findings from the survey and FOI requests support this, highlighting inconsistency in the organization and delivery of healthcare for pwALS in the UK. Of the 49 organizations that reported that they held specialist ALS clinics, there was no standard frequency, with clinics varying between weekly and six-monthly, with 50% providing these at 2–3 month intervals and 29% being tailored according to clinical need.

#### ALS dietetic services

Regarding specialist dietetic care, although 89 organizations (80%) identified that they had dietitians working generically in their organization, only seven (18%) stated there was an ALS-specific funded dietitian in the MDT. During the focus groups, this variability in care was a particularly emotive topic, with reports of patients being inappropriately declined dietetic input or being removed from a dietitian’s caseload due to not meeting “hard” referral criteria (e.g. greater than 10% weight loss). This, combined with long waiting times, was seen as a key reason why pwALS were denied timely dietetic input. Many dietetic participants felt that they saw pwALS when it was too late. Again, survey responses support this, suggesting that only 31% of pwALS were referred to a dietitian at diagnosis.

#### Setting of ALS care delivery

The FOI requests identified that ALS care is delivered in various settings, including out-patient clinics, in-patient services, community clinics, domiciliary visits, and hospice care, with one organization using video-conferencing. The value and utility of seeing patients in their home environment to achieve an accurate understanding of how they are managing their nutrition was also highlighted during focus groups.

#### Nutrition knowledge and skills

Another element of quality care was the knowledge and skills of HCPs. Most non-dietetic survey respondents (87%) said that they provided nutritional advice to pwALS. Within the focus groups, nurse specialists were reported to play a pivotal role in nutritional management. The importance of HCPs' skills and knowledge to practice effective ALS-specific nutrition management was also highlighted. Yet, there were mixed opinions about how well informed HCPs were and it was noted that the pre-gastrostomy period was the least well managed.

Just over 40% of survey respondents reported that they did not base their advice on guidelines or standards. Those that did referred mostly to the MNDA, NICE, PENG, ESPEN, and BAPEN. Many Trusts drew up their own guidance. The lack of ALS specific guidance for HCPs was cited as contributing to the variability in knowledge and practice in the focus groups. Participants stated that more education and formal guidance would help less knowledgeable professionals and facilitate the delivery of more standardized and effective nutritional management.

The importance of building relationships with pwALS was raised in every focus group, as well as the benefit of engaging patients at an early stage to facilitate high-quality nutritional management.

#### Commissioning of ALS services

Due to the complexities of achieving good nutritional care for people with ALS, there was a clear sense from focus group participants that one of the most important issues in successful nutritional management was the organizational and working approach adopted by both HCPs and commissioners.

A host of issues with commissioning were raised in each focus group, mostly relating to a perceived lack and inequality of specialized commissioning of nutrition management across areas, resulting in a “postcode lottery.”

Trying to understand how nutritional management services for pwALS are commissioned was a challenge across all parts of the study. Focus group participants described the commissioning for ALS as “illogical,” as it failed to recognize the time consuming nature of managing complex patients. This was reported to be particularly problematic for some dietitians, who were unable to allocate sufficient time to allow them to deal with the complex nutritional management of pwALS since their commissioning stipulated that they should spend 10–15 minutes in total with each patient. Dietitians reported facilitating the nutritional management for pwALS by practicing “good will” and opting to spend more time with them than they are commissioned for. Almost half of survey respondents believed that these services were given low (40.6%) or very low (6%) priority by commissioners. In general, there was a feeling from several focus group participants that, with better and more specific guidance about the need for nutritional management services in ALS, these services would be more likely to be commissioned

#### Organization and multidisciplinary team working

One of the most prominent topics raised in every focus group was the importance of implementing a multidisciplinary approach in order to deliver effective nutritional management.

Responses from the FOI request and survey suggested that the majority of organizations (73.2%) have an MDT. Although a dietitian was considered a member of the ALS MDT in 56 (79%) of organizations, only seven reported having ALS-specifically funded dietetic posts. Involvement in the MDT by all healthcare professions typically included attending regular meetings, whilst just over one quarter (26.2%) attended specialist ALS clinics.

One of the most important aspects of high functioning MDTs was effective communication between ALS professionals. Responses from the survey suggest that around half (52%) of HCPs think that communication about nutritional management is very or extremely effective. Furthermore, only 39% (*n* = 109) believe that care is very or extremely well-coordinated.

## Discussion

The complexity of providing nutritional care to pwALS has been acknowledged throughout this study. Against this background, our research has identified a number of factors that influence the national delivery of nutritional management services for pwALS.

Organization of care has been identified as a key consideration in delivering quality healthcare to pwALS (14). Despite this, our study highlights geographical differences in ALS services and dietetic provision across the country. Whilst meeting the needs of the local community undoubtedly requires flexibility in healthcare delivery, the extent to which participants in this study reported inequities was apparent.

Taking a MDT approach was highlighted in this study as being central to coordinated health services, the benefits of which are known to positively impact on the quality of care and subsequent outcomes for pwALS (20–22). Although findings from our study suggest that the majority of organizations have an ALS MDT, the configuration and function varies. A major issue here is that for most HCPs in the MDT, providing healthcare to pwALS is only part of a much wider caseload. Only a small number of staff work in an ALS dedicated role. This is also the case for dietitians whose work is pivotal in providing more specialist nutritional care. Given the complex nature of the nutritional problems that pwALS experience, there is a case for having more specialist and advanced dietetic practitioners working in this area.

Another important aspect here is that whilst it seems that most HCPs provide nutritional advice to pwALS, in many cases, this is not standardized or driven by evidence-based guidelines. Furthermore, as a large proportion of individuals felt that their ALS nutrition knowledge could be improved, our findings emphasize a need for ALS-specific nutrition education and training, especially for non-specialist HCPs (non-dietitians or those outside of ALS MDTs). For Allied Health Professionals, reviewing current practice against the competency framework for progressive neurological conditions would be an ideal starting point to identifying continuing education and development needs for individuals and teams (23).

Finally, the timeliness of nutritional intervention is important. Despite nutritional issues often being present at diagnosis, our findings suggest that less than a third of pwALS are able to access dietetic services at this time. Given that the majority of MDTs report having a dietitian included, the reasons why many pwALS have to wait much longer to access specialist nutritional assessment and advice requires further exploration. Due to the nature of the disease, lack of timely intervention, alongside rapid progression of symptoms that impact on nutritional intake, is likely to contribute to deterioration in nutritional status. As highlighted previously, evidence suggests that pwALS who avoid losing weight during the course of the disease live longer (3). As recommended in national guidance (14), if pwALS are to have their nutritional status assessed, managed, and reviewed at each stage of the disease course, a review of staff resources is essential to facilitate more timely nutritional intervention. Particularly in light of the recent global pandemic and consequent impact on health care delivery, wider use of remote care using novel technology, such as video-conferencing, should also be explored (24).

Central to the development of specialist services for pwALS is funding and commissioning. Our findings suggest that, for people working in UK MND services, this process is unclear. It is likely that this confusion, particularly with regards to nutrition services, acts as a barrier to addressing the inequity in provision of nutritional care of pwALS.

### Strengths and limitations

To our knowledge, this is the first study to extensively explore the national delivery of nutritional management services to pwALS. Using a three-part mixed-methods approach, we have been able to gain a deeper insight into how the variability in services impacts on HCPs working with pwALS across the country. Furthermore, given the number and geographical spread of participants that took part, we believe that we have captured a national snapshot of the structure of nutritional management services in the UK. There were, however, some gaps in our data. Although response rate to the FOI requests was high, the level of data collected varied considerably between organizations due to information not being available.

### Implications for practice

Although this study focused on the provision of nutritional management services for pwALS in the UK, we believe that many of the overarching themes that we identified are likely to be paralleled in other countries. Key action points for practice have been summarized in Table 6. While research is required to chart the structure of dietetic services for pwALS in other countries, these action points may contribute to the development of high quality nutritional management services for pwALS globally.

**Table 6:** Action points for practice.

Theme	Action point	Purpose
Access to dietetic services	Setting of national minimum standards for nutritional management of pwALS, including referral to dietetic services at diagnosis	To drive commissioning and timely, equal access to services
Organization and multidisciplinary team working	Local review of the MDT membership with the inclusion of a dietitian as a core member	Increased number of specialist and advanced dietetic practitioners working with pwALS
	Local evaluation of MDT communication and coordination of care, from the healthcare professional and patient perspective	To optimize the effectiveness of MDT working, with an overall goal of improving outcomes for pwALS
Nutrition knowledge and skills	Development of evidence based national nutritional guidelines specifically for pwALS, from diagnosis and throughout the disease course	To improve the standard of nutritional care for pwALS with increased awareness of the benefits of providing nutritional support
	National coordination of ALS-specific nutrition education and training, developed from the scientific evidence base, aimed primarily at non-specialist HCPs (non-dieticians or those outside of ALS MDT)	To ensure all healthcare professionals are providing evidence based nutritional advice to pwALS

## Conclusions

Our findings suggest that the development of evidence-based national guidelines for nutritional management in ALS, particularly at the time of diagnosis and pre-gastrostomy, could drive standardization of high-quality nutritional care, reduce inequities in services, and inform further nutrition education and training for HCPs. Furthermore, we believe that to reduce geographical variability, there is a need to improve understanding and transparency of the commissioning process of nutritional services for ALS in the UK.
